# Electrochemical Analysis of Polymer Membrane with Inorganic Nanoparticles for High-Temperature PEM Fuel Cells

**DOI:** 10.3390/membranes12070680

**Published:** 2022-06-30

**Authors:** DongWoong Choi

**Affiliations:** Department of Chemical Engineering, Dong-Eui University, Busan 47340, Korea; choidw@deu.ac.kr; Tel.: +82-51-890-1462

**Keywords:** ZrO_2_, TiO_2_, inorganic nanoparticle, polymer membrane, fuel cell

## Abstract

In order to solve the challenge that battery performance rapidly deteriorates at a high temperature condition of 100 °C or higher, ZrO_2_-TiO_2_ (ZT) with various Zr:Ti ratios synthesized by a sol-gel method were impregnated in a Nafion membrane. Through material characterization, a unique ZT crystal phase peak with a Zr-O-Ti bond was identified, and the band range associated with this bond and intrinsic functional group region could be identified. These prepared powders were blended with 10% (*w*/*w*) Nafion-water dispersion to prepare composite Nafion membranes (NZTs). The water uptake increased and the ion exchange capacity decreased as the TiO_2_ content increased in the NZTs in which particles were uniformly distributed. These results were superior to those of the conventional Nafion 112. The electrochemical properties of all membranes was measured using a polarization curve in a single cell with a reaction area of 9 cm^2^, and the operating conditions in humidified H_2_/air was 120 °C under 50% relative humidity (RH) and 2 atm. The composite membrane cell with nanoparticles of a Zr:Ti ratio of 1:3 (NZT13) exhibited the best electrochemical characteristics. These results can be explained by the improved physicochemical properties of NZT13, such as optimized water content and ion exchange capacity, strong intermolecular forces acting between water and nanofillers (δ), and increased tortuosity by the fillers (τ). The results of this study show that the NZT membrane can replace a conventional membrane under high-temperature and low-humidity conditions. To examine the effect of the content of the inorganic nanomaterials in the composite membrane, a composite membrane (NZT-20, NZT-30) having an inorganic nano-filler content of 20 or 30% (*w*/*w*) was also prepared. The performance was high in the order of NZT13, NZT-20, and NZT-30. This shows that not only the operating conditions but also the particle content can significantly affect the performance.

## 1. Introduction

Polymer electrolyte membrane fuel cells (PEMFCs) have attracted much attention in the past several decades because they have the highest power density of all the fuel cell classes, zero harmful emissions, a relatively low weight, and simple operation [1,2,3,4]. This makes them suitable for various applications such as vehicle, power generator, and uninterrupted power supply. The PEMFCs have many components, and one of them, the polymer electrolyte membrane serves as a hydrogen ion conductor and serves to separate the anode and cathode. Currently, the most widely used PEM is Nafion, which DuPont has manufactured since the 1960s. Nafion is an aliphatic compound composed of carbon and fluorine with a perfluorosulfonic acid (PFSA) structure. It consists of a side chain containing a sulfonic acid group on the main chain of a perfluorinated alkyl compound in which fluorine is substituted for hydrogen in the hydrocarbons. It has the properties of high hydrogen ionic conductivity, as well as good chemical and mechanical stability based on its unique chemical structure. The effect of temperature conditions on cell voltage was reported by a simulation program study [5]. To achieve the best performance, the FC with a Nafion membrane is usually operated at ~80 °C. However, this membrane has the shortcomings of high cost, an environmental issue regarding perfluorinated compounds, and the necessity to maintain constant water content to sustain a certain amount of proton conductivity.

The proton conductivity of Nafion is sensitive to dehydration, and its ability significantly decreases when the temperature is higher than 100 °C (the boiling point of water). Moreover, its mechanical and thermal stability is reduced under these operating conditions; therefore, this conventional Nafion membrane cannot be applied at temperatures of 100 °C. However, if the operating temperature range for a PEMFC application could be expanded to over 100 °C, there would be many advantages. First, its tolerance to impurities in the fuel gas could be enhanced. The possibility of a poisoning effect of platinum (Pt)-based anode catalysts by a trace amount of carbon monoxide (CO) present in the modified fuel gas would be lower [6,7]. Second, the electrode kinetics could be enhanced. The distances between molecules would become farther, which could help intermolecular collisions to occur more vigorously; for this reason, the kinetics of both electrodes can be faster at high temperature. 

Water management can be easier. Above its boiling point of 100 °C, water can exist in only one single phase (vapor); therefore, the management system could be simplified and a heat exchanger could be smaller. The heat generated can be supplied in various ways, such as direct heating, pressurized operation, and steam reforming. Heat exchangers can be made simpler and smaller than ones operating at 80 °C. Due to the advantages given above, many PEMFC studies have been conducted under high temperature and low humidity conditions.

Among the various studies mentioned above, one recently attracted attention which prepared a composite membrane by adding hygroscopic inorganic fillers (ZrO_2_, TiO_2_, SiO_2_, SO_4_^2−^/ZrO_2_, ZrP) to organic PFSA polymers [8,9,10,11,12,13]. SiO_2_ and TiO_2_ are known as hygroscopic materials that possess the ability to absorb large amounts of moisture; thus, these kinds of materials can be possible candidates for inorganic fillers with hydrophilicity. The modified PFSA membrane containing inorganic particles has been demonstrated to improve water retention and hydrogen proton conductivity at high temperatures; this is because of the effect of a specific interaction made possible by the incorporation of the organic/inorganic materials. Thus, those materials could enhance the performance of a fuel cell at high temperature when used as filler to a conventional Nafion membrane, even though these materials caused lower conductivity. These results indicate that the factor of water retention has more influence on increased performance than conductivity does under harsh operating conditions. The impregnating material (SO_4_^2−^/ZrO_2_ or ZrP) has been applied to increase the proton conductivity of a modified membrane at high temperatures. The material of a solid-state superacid, SO_4_^2−^/ZrO_2_ depends on the S/Zr ratio, and it has higher conductivity with a high S/Zr ratio. The proton conductivity of this sample was reported to be 2.3 × 10^−1^ S·cm^−1^ at 105–135 °C [14]. The SO_x_ bonded tightly with Zr on the surface of heat-treated SO_4_^2−^/ZrO_2_ induced electronic polarization. The electrons sites on O of SO_x_ and the Lewis acid sites on Zr are proposed to create new Brønsted acid sites, which would lead to high proton conductivities. The glass type of ZrP showed protonic conductivity of 10^−2^ S·cm^−1^ at room temperature under conditions of full humidification, and the reason was that the glassy phosphates created by the sol-gel method have hydrogen-bonded protons and large amounts of molecular water. Moreover, the mobility of protons in zirconium phosphate gel glasses is higher than that of alkali ions in other oxide matrices [15].

This study focuses on the electrochemical effect of a composite membrane, including inorganic fillers, on the PEMFC under the operating conditions of high temperature and low humidity. As inorganic nano-fillers, ZrO_2_-TiO_2_ nanoparticles with various Zr:Ti ratios (=1:3, 1:1, or 3:1) were synthesized by the sol-gel method. Those particles were incorporated into a Nafion membrane, and Nafion/ZrO_2_-TiO_2_ composite membranes with different Zr:Ti ratios were fabricated. The properties of the ZrO_2_-TiO_2_ particles were determined by XRD, and FT-IR. Tests of the water uptake and ionic exchange capacity (IEC) of the composite membranes were conducted to examine the physicochemical properties of the Nafion/ZrO_2_-TiO_2_ composite membranes and the performance of cells that included those composite membranes was tested. As control groups, commercial Nafion 112, Nafion/ZrO_2_, and Nafion/TiO_2_ were also prepared to compare with the composite membranes. Nafion/ZrO_2_-TiO_2_ membranes with Zr:Ti ratio of 1:3, 1:1, and 3:1 were denoted as NZT13, NZT11, and NZT31. Nafion 112, Nafion/ZrO_2_, and Nafion/TiO_2_ were referred to as N112, NZ, and NT.

This study focused on the relation between the electrochemical properties according to various Zr:Ti ratios (=1:3, 1:1, 3:1) in the binary oxide nanomaterials. This research also focused on finding the optimal property of the polymer composite membrane based on the optimal molar ratio. To the best of my knowledge, no studies have been conducted on the electrochemical analysis of various Zr:Ti ratios (Zr:Ti = 1:3, 1:1, 3:1) on NZT in polymer electrolyte membrane fuel cells. If so, this would be the first research with regard to this experimental exploration.

## 2. Experimental

### 2.1. Synthesis of ZrO_2_-TiO_2_

Fine ZrO_2_-TiO_2_ was prepared using the sol-gel method, as reported by Zou et al. [16]. Titanium chloride (TiCl_4_) or zirconium oxide chloride (ZrClO_2_) was dissolved in distilled water. Ammonium hydroxide (NH_4_OH) was added to this solution and titanium hydroxide (Ti(OH)_4_) or zirconium hydroxide (Zr(OH)_4_) was made to precipitate. The precipitate was filtered using aspirator and washed in distilled water several times while stirring until chlorine ions were completely eliminated. The nitrate solution was prepared by re-dissolving the titanium hydrous precipitate or zirconium hydrous precipitate in nitric acid with stirring. The solutions were mixed together at Zr:Ti ratios of 1:3, 1:1, and 3:1. Then ammonium hydroxide solution was added to adjust the mixed solution to pH 8. The solution was refluxed, and precipitates were formed at the end. The products were dried for 12 h; then, 0.25 M H_2_SO_4_ solution (15 mL/g of the sample) was added to these samples under vigorous stirring for 1 h and filtered using filter paper. The samples were calcined at 700 °C for 6 h. A ball-mill instrument (INTEC, UBM-100S, Seongnam, Korea) was used for 48 h to grind the samples into fine particles. In this way, the synthesized nanopowders were obtained. The nanoparticle of ZrO_2_-TiO_2_ with Zr:Ti ratio of 1:3, 1:1, and 3:1 were denoted as ZT13, ZT11, and ZT31.

### 2.2. Preparation of the Composite Membranes

A 10% (*w*/*w*) Nafion solution (DuPont, Wilmington, DE, USA) with a nominal equivalent weight (EW) of 1100 was used to prepare the Nafion composite membrane following the procedure related below. First, the Nafion solution was concentrated to 15% at 50 °C, and the synthesized powders were dispersed in isopropyl alcohol (IPA) in an ultrasonic bath for 15 min. Then, they were mechanically mixed with the concentrated Nafion solution for 30 min. The suspension was cast on a glass plate using the Doctor Blade coating method, and sequentially heated at 40 °C for 1 h and at 150 °C for 2 h. After a heat treatment process, the composite membranes were detached from the plates. To remove impurities within the membranes, the prepared composite electrolyte membranes were immersed in distilled water for 10 min, and then transferred to a 5 wt.% hydrogen peroxide (H_2_O_2_) solution and heated at 80 °C for 1 h. Then, the membranes were immersed in 1 M sulfate (H_2_SO_4_) at 80 °C for 1 h and then placed in water at 100 °C for 1 h. These processes of impurity removal were repeated twice.

### 2.3. Preparation of the Membrane Electrode Assemblies (MEAs)

The catalyst inks for the electrodes were prepared by mixing amounts of the appropriate 40% (*w*/*w*) Pt/C (E-Tek Inc.), 10 wt% Nafion solution (Dupont), and IPA (Sigma-Aldrich, HPLC grade, St. Louis, MO, USA). Ultrasonics were applied for 10 min for uniform dispersion. The catalyst inks were then sprayed on a composite membrane to provide the same platinum (Pt) loading (0.4 mg cm^−2^) on the anode or the cathode. The MEAs were prepared by drying for 4 h at 50 °C after the spray coating. SGL carbon papers were used with a thickness of 400 microns (GDLs, Sigracet carbon group, Meitingen, Germany).

### 2.4. Physical and Chemical Characterization

The water uptake (*W*_up_%) of the membranes was calculated by the following procedure. The wet weight (*W*_wet_) was measured after soaking a membrane sample in water at room temperature for 12 h. The sample was then dried in a vacuum oven at 100 °C for 2 h, and the dry weight (*W*_dry_) of the sample was measured. The weights obtained for each sample were substituted into Formula (1), and the amount of water uptake was calculated:

(1) $$W_{\mathrm{up}}\%=\frac{W_{\mathrm{wet}}-W_{\mathrm{dry}}}{W_{\mathrm{dry}}}\times 100$$

where [*W*_up%_] is the percentage of the water content absorbed in the sample, [*W*_wet_] is the wet weight (g), and [*W*_dry_] is the dry weight (g). The ion exchange capacity (IEC) defines the number of milli-equivalents of ions in 1 g of a dry membrane and the IEC of the membranes was measured using acid–base titration. A dry membrane was immersed in 1 M NaCl solution for 12 h at room temperature. To neutralize the exchanged proton (H^+^), this solution was titrated with a 0.01 M NaOH solution. The titration volume was followed by the pH variation and the amount of titrant used at the equivalence point were determined. The IEC was calculated using Formula (2):

(2) $$\mathrm{IEC}=\frac{V\times M}{m}$$

where [IEC] is the ion exchange capacity (meq·g^−1^), [*V*] is the titrant volume added at the equivalence point (mL), [*M*] is the molar concentration of the titration, [*m*] is the dry sample mass (g). The structure of the samples was analyzed using X-ray diffraction (XRD, Philips XPRET MPD, Amsterdam, The Nederlands) with Cu-Kα1 radiation, and the 2θ angles were scanned from 5 to 85°. The microstructure and morphology in all membranes were observed using field-emission scanning electron microscopy (FE-SEM, Hitachi S-4300, Tokyo, Japan). The synthesized samples were observed by Fourier transform infrared spectroscopy (FTIR, Bomem DA8, Québec, Canada). The FT-IR spectra were recorded in the wave number range from 4000 to 500 cm^−1^. A particle size distribution (PSD) test was performed to measure the size of the prepared powder. A solution prepared by dispersing nanoparticles in distilled water was transferred to an empty quartz cell using a pipette. The particles in this cell were tested by the dynamic light scattering method on a Zeta Potential and Particle Size Analyzer (ELSZ-2000 Series, Otsuka, Tokyo, Japan).

### 2.5. Electrochemical Characterization

Electrochemical tests were conducted in a single cell with a reaction area of 9 cm^2^ under humidified H_2_/Air conditions. The operating temperature was 120 °C, 50% RH. When operating at 120 °C, the pressure of inlet gases was 2 atm to prevent dehydration of the membranes. For NZT13 with different inorganic ZT content, the experimental conditions at 120 °C and 30 or 10% RH were added. The gas flow rates were fixed at 1.5 times the stoichiometric value of the fuel and two times the stoichiometry of the oxidant. The polarization curve was obtained by supplying a constant current to each point for 3 min in an electron loader (Daegil Electronics, EP-1000, Gyeongangnam-do, Korea).

## 3. Results and Discussion

**Figure 1:**
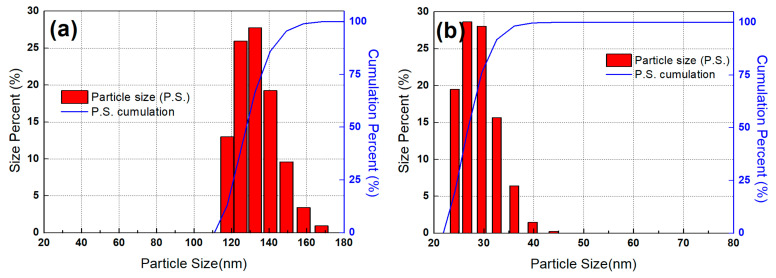
Particle size distribution of ZrO_2_-TiO_2_ (**a**) before and (**b**) after ball milling.

Various polymer electrolyte composite membranes were prepared, and their physical properties are summarized in Table 1. The particle size distribution (PSD) was conducted to determine the size of the prepared powders. Figure 1 shows the PSD of the ZrO_2_-TiO_2_ binary oxide with Zr:Ti ratio of 1:3 (a) before and (b) after the process of ball-milling for 48 h. The average particle size was reduced from 139 to 29 nm through the ball mill operation. The wide PSD was also changed to a narrower distribution within a certain range. The powders before ball milling had relatively large particle size and wide PSD, and these properties would make it difficult to disperse the powders uniformly in the Nafion polymer matrix. On the other hand, the particles after ball milling showed a smaller size and narrower PSD, which allowed homogeneous dispersion of the samples in the Nafion.

**Figure 2:**
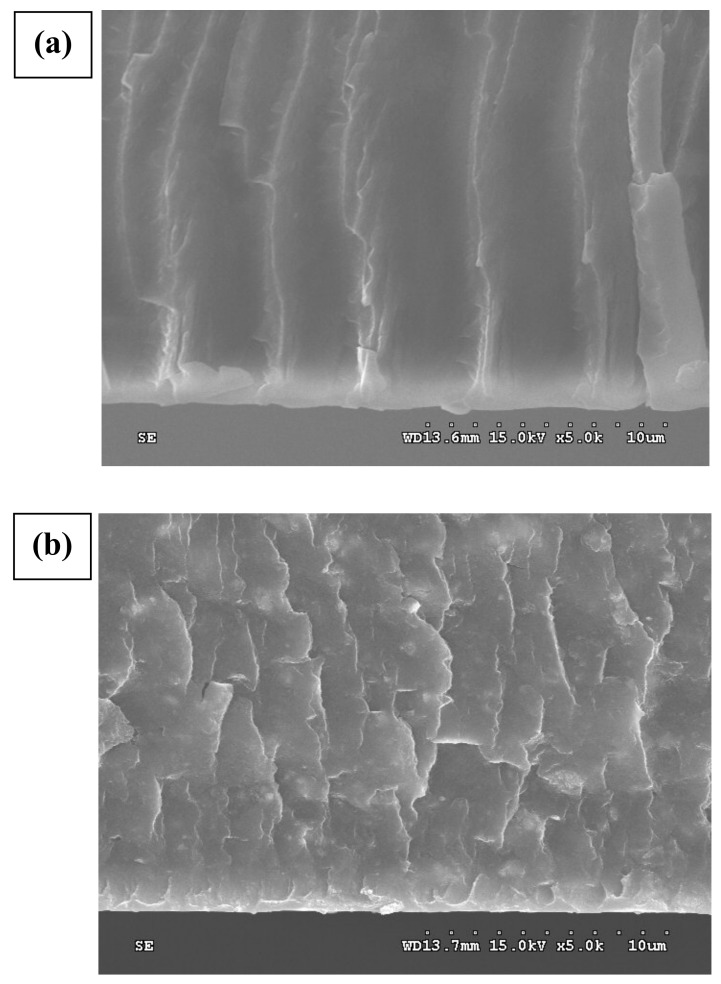
Cross-sectional SEM images of (**a**) Nafion 112, (**b**) NZT13 (Zr:Ti = 1:3).

The cross-section of the Nafion and NZT13 (Zr:Ti = 1:3) membrane was analyzed using FE-SEM at the same magnifications. Figure 2a,b illustrates a cross-section image of the Nafion electrolyte membrane and of the composite electrolyte membrane NZT13, which were magnified by 5000 times. No white particles were observed in the images (a) of the Nafion membrane. However, in the case of NZT13, the synthesized inorganic nanoparticles were clearly shown as white spots and these spots were evenly distributed in the Nafion matrix. The cross-sectional images of the pristine Nafion 112 presented almost straight linear lines. The linear lines are thought to be related to ‘-[CF_2_-CF_2_]_n_-[CF_2_-CF]_m_-’ of the main PTFE chains in Nafion polymer composed of main chains, side chains, and sulfone end groups. Herein, the subscript “n, m” denotes the degree of polymerization, that is, the number of units linked together. However, the images of the composite membrane showed highly branched lines. The linear lines were transformed into a more complex network of cross-section lines by the inorganic filler. This may demonstrate that the composite polymers were more tortuous by containing the inorganic nanoparticles in the pristine polymers.

**Figure 3:**
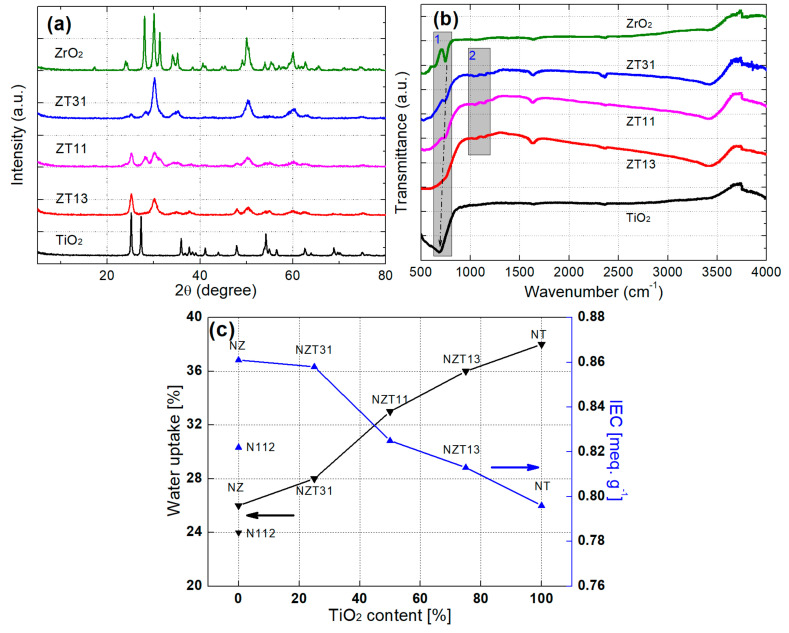
(**a**) X-ray diffraction patterns; (**b**) FT-IR spectra of ZrO_2_, ZrO_2_-TiO_2_ with different Zr:Ti ratios (Zr:Ti = 3:1, 1:1, and 1:3) and TiO_2_ particles; (**c**) Water uptake and ion exchange capacity (IEC) of N112 and the composite membranes (NZ, NT, NZT31, NZT11, NZT13).

The structures of the synthesized ZT powders were analyzed by X-ray diffraction (XRD, Cu-Kα1 radiation). Figure 3a shows the results of XRD analysis of ZT composite inorganic particles with different Zr:Ti ratios. As a control group, the pristine ZrO_2_ and TiO_2_ samples were also analyzed to compare the patterns of the peaks in the ZT samples. Diffraction peaks can be observed for all samples, which indicates the formation of crystalline structures. The XRD patterns of the three ZT samples show a strong, unique peak of ZrTiO_4_ at circa 2θ = 30.7° [16]. This might be a peak unique to the crystal ZT phase with Zr-O-Ti bonds. The peak at 2θ = 25.1 is shown for TiO_2_ and ZT samples, and the intensity of this peak decreases with a reduction in the Ti ratio from ZT13 to ZT31. No peak at circa 2θ = 25° was detected for the ZrO_2_ sample. For ZrO_2_ and ZTs, the peak intensity occurs at 2θ = 50.0° and this intensity declines with decrease in the Zr ratio of the samples. Finally, no peak was observed for TiO_2_. The change in this intensity could be explained as follows: the titanium ions of TiO_2_ (or the zirconium ions of ZrO_2_) were replaced with zirconium (or titanium) ions of the ZT. These results for the peaks indicated that ZrO_2_- TiO_2_ composite particles were successfully synthesized.

FTIR spectra were used to investigate the molecular bond structure of Zr-O-Ti in the synthesized ZT samples. The FTIR spectra of the ZrO_2_, TiO_2_, and ZT with different Zr:Ti ratios are presented in Figure 3b. Each peak of ZrO_2_, ZT31, and ZT11 can be observed at 746, 744, and 739 cm^−1^. In the case of ZT13 and TiO_2_, the band was seen at *circa* 714 or 683 cm^−1^. According to references, the peak in the region of 780–790 cm^−1^ could be ascribed to the asymmetric vibration of the Zr-O-Zr bond, and the band at 500–1000 cm^−1^ corresponded to the stretching vibration of Ti-O and Ti-O-Ti bonds [17,18]. Two things could be inferred from the results of these reports. When the Zr ratio gradually increased, the shape of the band related to TiO_2_ was changed to the peak associated with ZrO_2_, and Ti ions of the Ti-O-Ti bond in the band region of TiO_2_ was changed with Zr ions (gray rectangle 1). These results from the formation of a ZT network occur as the replacement of the Zr atoms proceeds. Therefore, these would be related to Zr-O-Ti bonds. Additionally, the unique small peaks at 1140 and 1050 cm^−1^ were presented in three ZT samples, except for the ZrO_2_ and TiO_2_ samples (gray rectangle 2). These frequencies might present unique functional groups associated with Zr-O-Ti bonds in ZT binary oxides.

The water content and ion exchange capacity (IEC) of the composite NZT membranes were measured, and the N112, NZ, and NT membranes were also measured to compare with the results of these membranes. The results of these experiments are summarized in Figure 3c. All NZT membranes containing inorganic nanoparticles were superior to the Nafion regarding water content, and the NZT31 and NZT11 also showed a greater increase in the IEC than the Nafion did. As the TiO_2_ content in the composite membrane increased, the capability for water uptake showed a tendency to enhance; however, the IEC showed a tendency to decrease. This means that the IEC improves with increasing ZrO_2_ content in the composite membrane. These suggested the relative effects of TiO_2_ and ZrO_2_ contents on the improvement of the water contents and IEC, respectively. Several studies have been reported on the Lewis acid and Brønsted acid sites of ZrO_2_-TiO_2_ and other materials containing Zr or Ti atoms [16,19,20]. Lewis acid sites increase as the Ti content increases, whereas the Brønsted acid sites increase as the Zr content increases. Because Lewis acid sites play a role in absorbing water molecules and Brønsted acid sites increase the proton exchange sites, when the content of Ti increases and the content of Zr decreases, the water uptake increases, and the IEC decreases. Therefore, it is important to add ZrO_2_-TiO_2_ to Nafion in an appropriate Zr:Ti ratio, and thus various composite membranes were prepared to find a composite membrane with an optimal IEC and water content. It was confirmed through the experiment that the use of TiO_2_ and ZrO_2_ in the binary oxides could improve the water uptake of the modified membrane, respectively.

**Figure 4:**
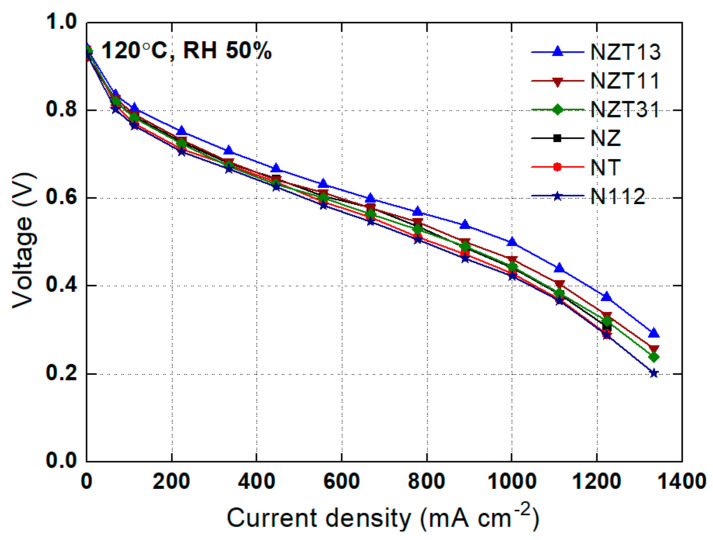
Polarization curves for membranes at 120 °C, RH 50%, 2 atm.

A test for polarization curve was conducted using the NZT composite membranes at the operating conditions of 120 °C, RH 50%, and 2 atm, and the results were compared with the results obtained using other membranes. These are shown in Figure 4. Overall, the performance of the composite membrane is superior to that of the Nafion membrane. NZT13 was the composite membrane with the best performance, while the NZT11, NZT31, NZ, and NT membranes show good performance in that order. There are two possible reasons for the good performance of the composite NZT13 membranes. The first is that physicochemical properties such as the water content and ion exchange capacity of the membrane were improved by impregnation with inorganic particles. In harsh operation of high-temperature and low-humidity, the composite membranes show good performance because the inorganic nanoparticles containing a lot of moisture everywhere help the ions to be transferred well. Therefore, a composite membrane with higher water content could help to improve cell performance. The cell with a composite membrane was yielded to have different cell voltage according to the content ratio of Zr:Ti, and the cell with NZT13 provided higher performance than the others. This is because a high Ti content in NZT13 generates substantial water uptake, which can minimize the phenomenon of membrane drying under the condition of high temperatures. Moreover, a certain amount of Zr in NZT13 improves the ion exchange capacity, which can be superior to composite membranes containing only Ti. The ZrTiO_4_ related to the synthesized ZrO_2_-TiO_2_ materials may support strong interaction between the membrane matrix and the absorbed water. As in a previous report [21], the high oxidizing potential material TiO_2_ results in the oxidation of water molecules and then leads to the formation of Ti-OH on the particle surfaces. Other OH groups add to the number of ion exchange sites in the modified membrane. Based on that report, ZrTiO_4_ may produce Ti-OH from TiO_2_, and also provide Zr-OH, one of the other OH groups, from ZrO_2_.

**Figure 5:**
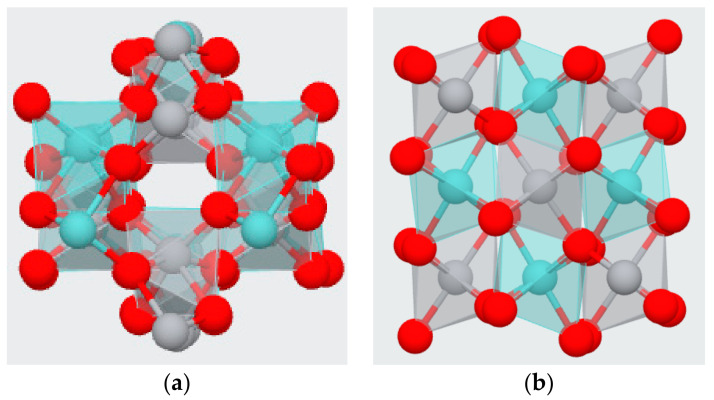
Schematic of two possible molecular structures (**a**,**b**) for ZrO_2_-TiO_2_ (ZrTiO_4_). (red, grey, or cyan ball indicates oxygen, titanium, or zirconium, respectively).

**Figure 6:**
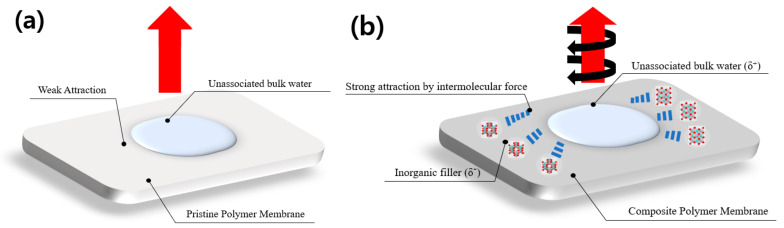
(**a**) Comparison of pristine polymer membrane and (**b**) composite polymer membrane with inorganic nanoparticles regarding an intermolecular force.

The possible molecular structure of ZrTiO_4_ is presented in Figure 5 below. There is a force that acts intermolecularly, and the greater this force, the more difficult it is to separate between the molecules. Oxygen atoms of ZrTiO_4_ or of OH groups formed from ZrTiO_4_ have high electronegativity, which can strongly attract hydrogen atoms of the unassociated bulk water molecules to form hydrogen bonds, and is illustrated in Figure 6. These bonds could make water sticky to the modified composite membrane. Therefore, modified ZT membranes based on ZrTiO_4_, which has a greater amount of water and a number of exchange sites available, exhibited high performance. Another explanation can be added here. Hydrogen cations are hydrated in water and can exist in the forms of H_3_O^+^, H_9_O_4_^+^, H_13_O_6_^+^, etc. This allows to create a more forceful positive charge than the hydrogen positive charge made by an over-concentration of electrons to an opposite side in neutral water. Abundant oxygen atoms of the nanoparticles filled in the membrane can form a strong oxygen negative charge. Therefore, stronger hydrogen bonds can possibly be induced between the water hydrated with hydrogen cations and the composite membrane filled with nanoparticles. The composite membranes that have these solid bonds can suppress water evaporation at higher temperatures than pristine membranes.

**Figure 7:**
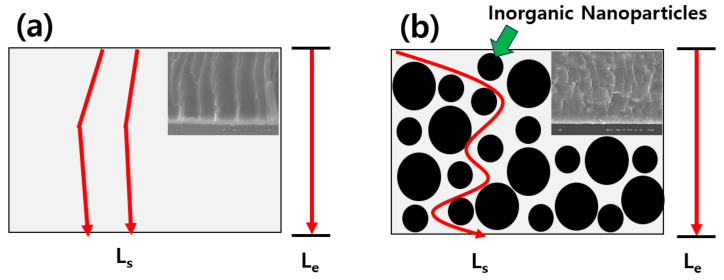
Comparison of geometrical tortuosity of (**a**) Nafion 112 and (**b**) NZT13 membranes (inset: the cross-sectional SEM images brought from Figure 2).

The second reason is that the gas crossover in the modified membranes may decrease because the tortuosity (τ) of the mass transfer pathways was increased by the structural change in the membranes. The tortuosity can be associated with “the average distance when a gas or fluid passes through the porous material (L_s_)” and “the thickness of the porous material (L_e_)”. The process for embedding the inorganic ZT nanoparticles in Nafion increases the Ls ratio. This explanation is shown in Figure 7, and this could also correlate with the results of the cross-sectional SEM images in Figure 2. NZT13 may have a higher L_s_ ratio and ‘τ’ than the others, and could minimize gas crossover during operation, which would lead to good performance.

**Figure 8:**
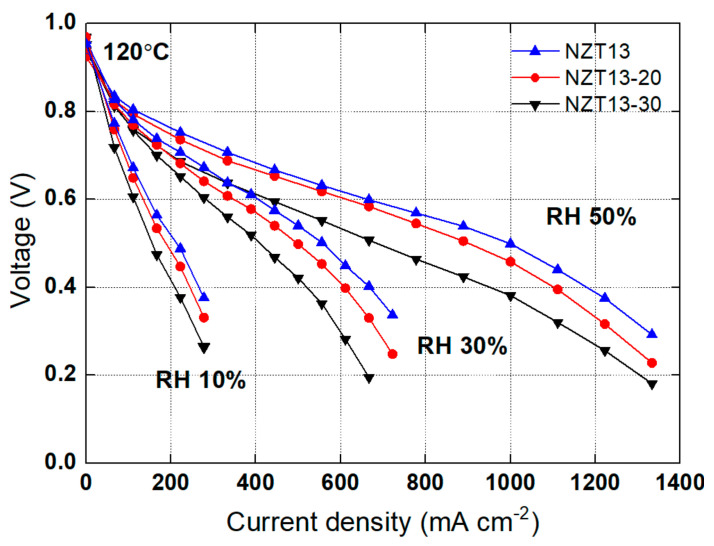
Polarization curves for the NZT13 with different inorganic ZT content (10, 20, and 30%) at 120 °C, RH 50, 30, 10%, and 2 atm.

To examine the effect of the content of the inorganic nanomaterials in the composite membrane, a composite membrane having an inorganic nano-filler content of 20 or 30% (*w*/*w*) was also prepared. The polarization curve was compared with that of the NZT13 membrane containing a nanoparticle content of 10%. These prepared samples were represented as NZT-20 and NZT-30. The measurements of the polarization curve were carried out at 120 °C, RH 10, 30, or 50%, and 2 atm, and the results are shown in Figure 8. As a result, NZT13, which has an inorganic nano-filler content of 10% (*w*/*w*), shows the highest performance under all conditions of humidity. The performance was high in the order of NZT13, NZT-20, and NZT-30. This shows that not only the operating conditions, but also the particle content can significantly affect the performance. As the inorganic nanomaterial content in a composite membrane increases, it becomes difficult to disperse the inorganic particles uniformly in the polymer matrix, and the inorganic particles aggregate easily. These would act to increase the resistance to hydrogen-ion conduction. Thus, it would finally lead to a decrease in performance. 

## 4. Conclusions

ZrO_2_-TiO_2_ nanoparticles (ZT) with various Zr:Ti ratios were synthesized, and a Nafion/ZrO_2_-TiO_2_ (NZT) composite membrane that can be applied under high temperature and low humidity conditions was fabricated. A unique peak on the crystal ZT phase with Zr-O-Ti bond, the band range related to this bond, and the peaks related to the intrinsic functional group of the ZT samples could be confirmed through XRD and FTIR analyses. The homogenous distribution of the inorganic nanopowders was verified in the polymer matrix by SEM, and the water uptake increased and ion exchange capacity decreased with increasing TiO_2_ content in the NZT membranes. The electrochemical properties of these membranes were examined using a polarization curve. The results were compared from a conventional Nafion (N112), the composite Nafion/ZrO_2_ (NZ), and the composite Nafion/TiO_2_ (NT) membranes. At high temperature and low humidity (120 °C, RH 50%, 2 atm), the polarization curve of the NZT13 membrane showed superior properties to that of the other membranes. These are due to the optimized water content and ion exchange capacity, the strong force (δ) that mediates the interaction between molecules, and the increased tortuosity (τ) caused by the impregnation of the nanoparticles to the Nafion membrane. Such combined features served to provide good electrochemical properties. As NZT13 showed better electrochemical properties than NZT31, it can be seen that water uptake is a more important factor for high-temperature performance than ion exchange capacity. The results of this study showed that embedding ZrO_2_-TiO_2_ nanoparticles in a membrane could address the issues that occur when a conventional membrane is applied to a PEMFC under high temperature and low humidity. To examine the effect of the content of the inorganic nanomaterials in the composite membrane, a composite membrane (NZT-20, NZT-30) having an inorganic nano-filler content of 20 or 30% (*w*/*w*) was also prepared. The performance was high in the order of NZT13, NZT-20, and NZT-30. This shows that not only the operating conditions but also the particle content can significantly affect the performance.

## Figures and Tables

**Table 1:** Type of membrane used in this experiment.

Membrane	Inorganic Nanoparticles	Content of Inorganic Nanoparticles (%)	Water Uptake	Thickness (μm)
Nafion 112	-	-	24	50
NZ	ZrO_2_	10	26	52
NT	TiO_2_	10	38	51
NZT31	ZrO_2_-TiO_2_ (Zr:Ti = 3:1)	10	28	52
NZT11	ZrO_2_-TiO_2_ (Zr:Ti = 1:1)	10	33	52
NZT13	ZrO_2_-TiO_2_ (Zr:Ti = 1:3)	10	36	52
		20	36	52
		30	37	52

## Data Availability

The data presented in this study are available on request from the corresponding author.
